# Influence of Grain Size and Its Distribution on Charpy Impact Properties of TA3 Alloy

**DOI:** 10.3390/ma15238537

**Published:** 2022-11-30

**Authors:** Chao Xin, Qi Wang, Junqiang Ren, Yonghong Zhang, Liang Zhang, Biao Sang, Le Li

**Affiliations:** 1 Xi’an Rare Metal Materials Institute Co., Ltd., Xi’an 710049, China; 2 School of Energy Engineering, Huanghuai University, Zhumadian 463000, China; 3 State Key Laboratory of Advanced Processing and Recycling of Nonferrous Metals, School of Materials Science and Engineering, Lanzhou University of Technology, Lanzhou 730050, China; 4 School of Materials Science and Engineering, Shaanxi University of Technology, Hanzhong 723001, China

**Keywords:** grain size, Charpy impact, Ti alloy, twin inducing plasticity

## Abstract

In practice, most components often receive impact loads during service. In order to ensure the service safety of components, impact toughness evaluation is essential. To the best of our knowledge, the previous studies were mainly focused on the quasi-static tensile deformation, and the impact toughness of bimodal grain structured metals have rarely been reported. Three different grain size characteristics TA3 alloy, i.e., fine grained sample (FG Ti), the mixture of coarse and fine grained sample (MG Ti), and coarse grained (CG Ti), were produced, and their tensile and Charpy impact properties were comparatively investigated. Owing to the strengthening of retained β phase and the twining inducing plasticity effect, MG Ti display the highest tensile strength and impact absorbed energy, together with an intermediate tensile elongation. The impact deformed microstructures revealed that the primary deformation modes of FG Ti, MG Ti and CG Ti sample are: dislocation slips, a combination of dislocation slip in fine grained region and $\left\{10\bar{1}2\right\}$ deformation twins in coarse grained region, and $\left\{11\bar{2}1\right\}$ deformation twins in sequence.

## 1. Introduction

Grain size is one of the crucial microstructural parameters that tailor the mechanical properties of the metals. Grain refinement is proposed an effective strategy to improve both strength and ductility of metals in the micrometer scale [1,2]. However, when the grain size was refined to sub-micrometer or less, such as the ultrafine-grained (UFG) or nano-grained metals produced by severe plastic deformation (SPD), their strength is significantly improved together with a decreased ductility [3,4,5,6]. Meanwhile, the grain size also has an influence on the deformation mechanism [7,8]. As the grain size decreases from micrometer to nanometer, the deformation modes change from the dislocations slip and/or deformation twins (DTs) to the grain boundary (GB)-mediated deformation mechanisms, such as GB sliding, grain rotation, or GB migration due to the limited dislocation activity in the nano-grains (NGs) and the high fraction of GBs [9]. For the hexagonal-closed packed (hcp) crystals, the frequency of the activated DTs is sensitive to the grain size [10,11]. As the grain size decreases to the sub-micrometer, the activities of DTs are gradually suppressed [12].

Apart from the grain size, the grain size distribution also has an obvious effect on the mechanical properties of metals. It has been proven that the metals with a bimodal distribution in grain size favored a desirable strength-ductility synergy [13,14]. Bimodal grain size distribution was introduced to NG metals to improve the ductility without much loss of their strength [15,16]. For instance, Wang et al. achieved high ductility in NG copper by embedding the micrometre-sized grains inside the NG and UFG matrix [17]. So far, bimodal grain structure has been successfully fabricated in different metals, such as Mg alloys [18,19], Mo alloy [20], Cu [21], 316 L stainless steel [22], TWIP steel [23], etc. Meanwhile, the formation mechanisms of bimodal grain structure [24,25] and the individual roles of fine and coarse grain as well as their synergy effect on mechanical properties were further discussed.

Based on the above description, one question could be proposed: What is the effect of grain size and grain size distribution on impact toughness and can the bimodal grain structure can also obtain improved impact toughness? To answer the above question, TA3 alloy is selected as a model in the present work, three different grain size distributions, i.e., fine grain size (FG Ti), coarse grain size (CG Ti) and a mixture of coarse and fine grains size (MG Ti), are produced by combing hot-rolling and annealing treatment. The effects of grain size and its distribution characterization of TA3 alloy on the tensile and Charpy impact are investigated, and the emphasis is placed on the deformation mechanism during impact deformation.

## 2. Experimental

A hot-rolled TA3 alloy plate with composition (in wt. %) of 0.25 Fe, 0.016 C, 0.02 N, 0.003 H, and 0.25 O was used in this study. The β transus temperature was determined to be 890 °C ± 5 °C by a TGA/DSC synchronous thermal analyzer. To obtain the different grain size distributions, the rolled plates were subsequently annealed at 870 °C, 900 °C, and 930 °C for 30 min and air cooling.

Tensile and Charpy impact specimens were cut from the annealed plates. Round tensile specimens with a gage diameter of 5 mm and a gage length of 30 mm were tested at room temperature at a constant cross-head speed of 1 mm/min by an Instron 5982 machine. The standard Charpy V-notch specimens with size of 10 × 10 × 55 mm were tested at the room temperature using an Amsler instrument with a capacity of 300 J and an impact speed of 5.23 m/s and the load and displacement of the hammer were recorded by a test Xpert data acquisition system. Three tensile and Charpy impact specimens of each condition were tested to ensure the experimental reproducibility.

The initial and deformed microstructures were observed using Olympus BX51M polarizing microscope, Quanta 450 scanning electron microscope (SEM) equipped with an electron back-scatter diffraction (EBSD) detector, and JEM-2100F transmission electron microscope (TEM). The metallographic samples were first mechanically polished and etched in a corrosive of H_2_O:HNO_3_:HF = 10:3:1 for 10 s, and then anodized in the sulfuric acid system. The EBSD samples were electro-polished in an electrolyte consisting of 10% perchloric acid and 90% acetic acid. The TEM foils were prepared by a twin jet electrochemical polishing device at about −30 °C and in an electrolyte consisting of 5% perchloric acid, 35% n-butyl alcohol, and 60% methanol.

## 3. Results

### 3.1. Initial Microstructure

**Figure 1:**
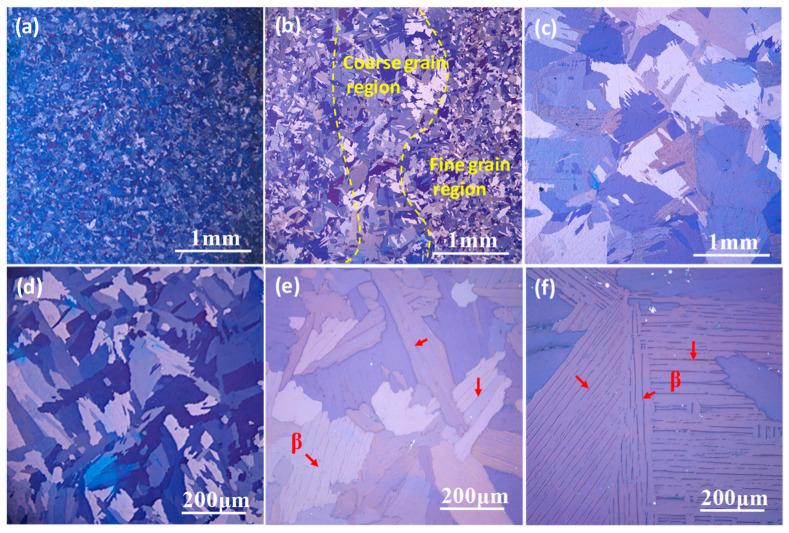
The initial polarized optical microstructures of TA3 alloy with different grain size characteristics. (**a**,**d**) are FG Ti, (**b**,**e**) are MG Ti, (**c**,**f**) are CG Ti.

The polarized optical microstructures of hot-rolling plate after annealing at different temperatures are shown in Figure 1. After annealing at 870 °C, the microstructure is characterized with the relatively fine grains (hereafter designated as FG Ti), and the grain shape is not typically equiaxed, as shown in Figure 1a,d. After annealing at 900 °C, a part of fine grains occur coarsening, where a mixture of coarse grains region and fine grains region is recognized (hereafter designated as MG Ti), as shown in Figure 1b. A further observation found a few acicular phases are distributed in the coarse grains, as indicated by the red arrows in Figure 1e. These acicular phases have been identified as retained β phase in the following section. When the annealing temperature is increased to 930 °C, its microstructure is typical coarse grain due to the occurring of abnormal grain growth (hereafter designated as CG Ti). Meanwhile, the density of retained β phase in CG Ti is relatively larger than that in MG Ti sample, as shown in Figure 1c,f.

**Figure 2:**
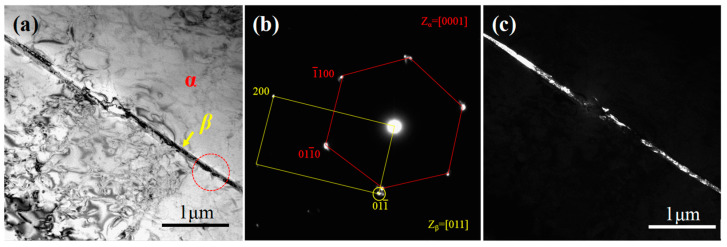
TEM images of the initial CG Ti sample: (**a**) light filed image, (**b**) select electron diffraction pattern, and (**c**) the corresponding dark filed image.

TEM technologies were carried out to identify the acicular phase in the matrix of Figure 1f. Figure 2a,c show the light field image and dark field image of the acicular phase with a thickness of 80 nm in the CG Ti sample. According to the diffraction pattern in Figure 2b, the interface between the α matrix and the acicular phase obeys a classical Burgers orientation relationship ${\left[0001\right]}_{\alpha }//{\left[011\right]}_{\beta }$ and ${\left(\bar{1}\bar{1}20\right)}_{\alpha }//{\left(1\bar{1}1\right)}_{\beta }$ [26,27]. Thus, the acicular phases in Figure 1e,f can be identified as the retained β phase.

**Figure 3:**
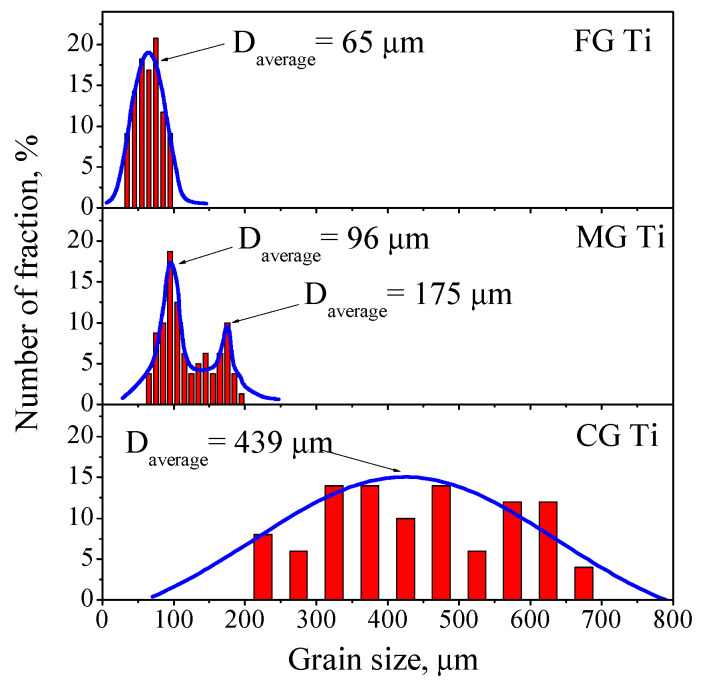
The grain size distributions of FG Ti, MG Ti, and CG Ti sample.

The grain size of the three different samples was statistically measured using Image-Pro software, as shown in Figure 3. It is clear that the grain size of the FG Ti and CG Ti samples are normally distributed and the average grain sizes are 65 μm and 439 μm, respectively. However, the grain size distribution of MG Ti sample presents a bimodal distribution, and the fine and coarse grain size are 96 μm and 175 μm, respectively.

### 3.2. Mechanical Properties

**Figure 4:**
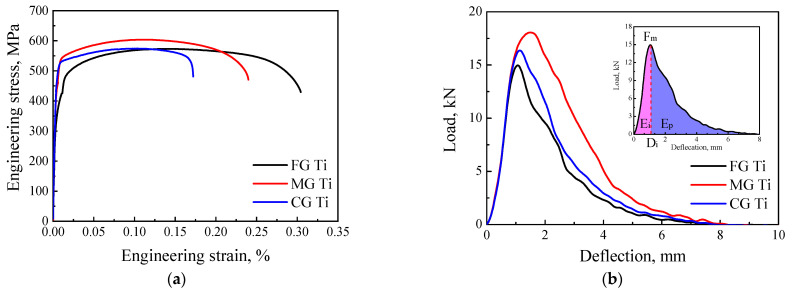

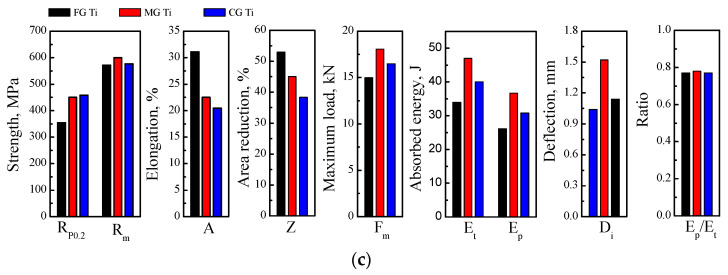
The mechanical properties of the FG Ti, MG Ti, and CG Ti samples: (**a**) tensile engineering stress-strain curves, (**b**) Charpy impact load-deflection curves, and (**c**) the tensile and Charpy impact properties of the three samples.

Figure 4a shows the tensile engineering stress-strain curves of the three samples. Table 1 displays the average yield strength (Rp_0.2_) and elongation at break (A) of FG Ti, MG Ti and CG Ti specimens. Among the three samples, the FG Ti samples manifest the lowest yield strength (Rp_0.2_) and the highest elongation to failure (A). The MG Ti samples display the highest Rp_0.2_ and ultimate tensile strength (R_m_) together with the intermediate A. The CG Ti samples exhibit the intermediate Rp_0.2_ and R_m_, but the lowest A.

Figure 4b illustrates the Charpy impact load-deflection curves of the three samples. As shown in the inset figure, the impact load firstly increased sharply and reached the maximum load (F_m_), which is defined as the crack initiation stage. Then the impact load gradually decreased until the sample breaks, which is defined as the crack propagation stage. Correspondingly, the impact absorbed energy (E_t_) can be divided into the crack initiation energy (E_i_) and crack propagation energy (E_p_) [28,29,30]. The comparison of the three samples demonstrate that, the MG Ti sample simultaneously exhibits the highest F_m_ and the biggest deflection of crack initiation (D_i_), which results in the highest E_t_. Figure 4c shows the tensile properties and Charpy impact properties of FG Ti, MG Ti, and CG Ti sample, respectively. It is concluded that the MG Ti sample exhibits the highest strength (including the Rp_0.2_ and R_m_) and impact absorbed energy (including E_t_, E_i_ and E_p_), simultaneously, together with the intermediate ductility (including A and Z).

### 3.3. Deformed Microstructures

**Figure 5:**
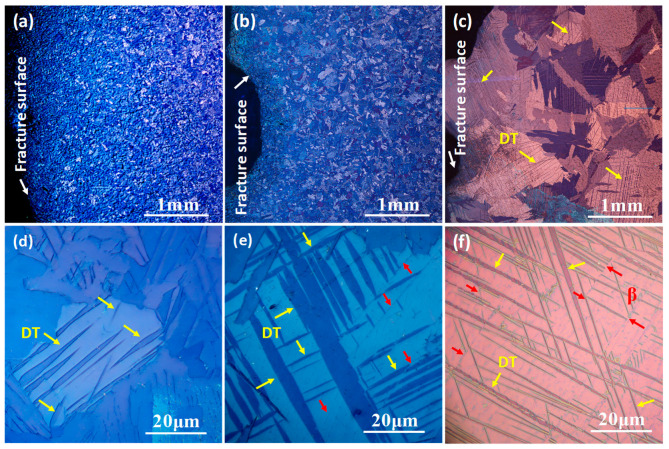
Polarized optical microstructures of TA3 alloy with different grain size characteristics after impact deformation. (**a**–**c**) are the low-magnification images near the fracture surface of FG Ti, MG Ti and CG Ti, respectively. (**d**–**f**) are the corresponding high-magnification image.

The polarized optical microstructures of TA3 alloy with different grain size characteristics after impact deformation are displayed in Figure 5. Compared to the initial microstructures (Figure 1), the low-magnification deformed microstructures near the impact fracture are blurred and rough, as shown in Figure 5a–c. Furthermore, the deformation twins (TBs) are observed in the high-magnification deformed microstructures, and their frequency is sensitive to the grain size characteristics. It can be found that the frequency of TBs gradually increases with the increasing of grain size, and the width of DTs in MG Ti is significantly larger than that in CG Ti. For FG Ti sample, the DTs mainly nucleated at GBs and grown inwards. For MG Ti and CG Ti samples, the intersection of different DTs was observed due to the simultaneous nucleation of DTs at GBs and α/β interfaces, as indicated by the red arrows in Figure 5e,f.

**Figure 6:**
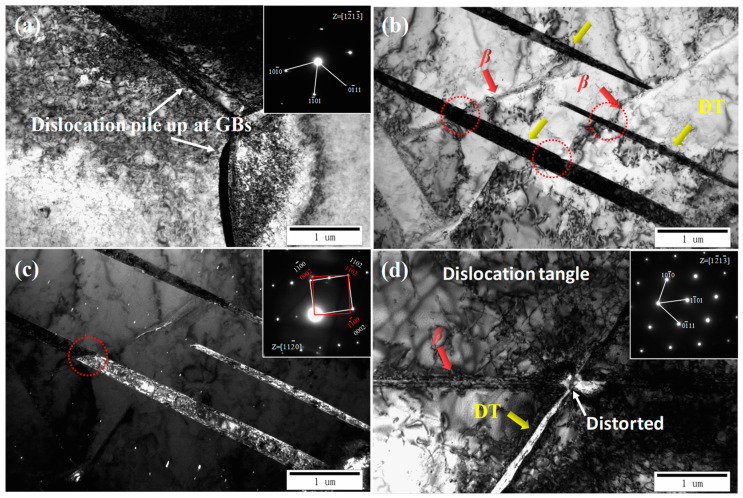
TEM images of the different TA3 samples after impact deformation, about 2 mm away from the impact fracture surface. (**a**) FG Ti, (**b**,**c**) MG Ti, (**d**) CG Ti, and the inset images are the corresponding select electron diffraction patterns.

Figure 6 demonstrates the TEM images of FG Ti, MG Ti and CG Ti sample after impact deformation. High density of dislocations was observed in the FG Ti sample and those dislocations piled-up at the GBs, as shown in Figure 6a. For the MG Ti sample, the parallel DTs were observed, and these DTs are identified to be $\left\{10\bar{1}2\right\}$ according to the selected area electron diffraction pattern. Meanwhile, the DTs can span the retained β lamellae, as indicated by the red circles in the Figure 6b. For CG Ti sample, the thin DTs and dislocation tangle were observed, and they were severely hindered by the retained β lamellae, resulting in the α/β interface to be severely distorted, as indicated by the arrows in Figure 6d.

**Figure 7:**
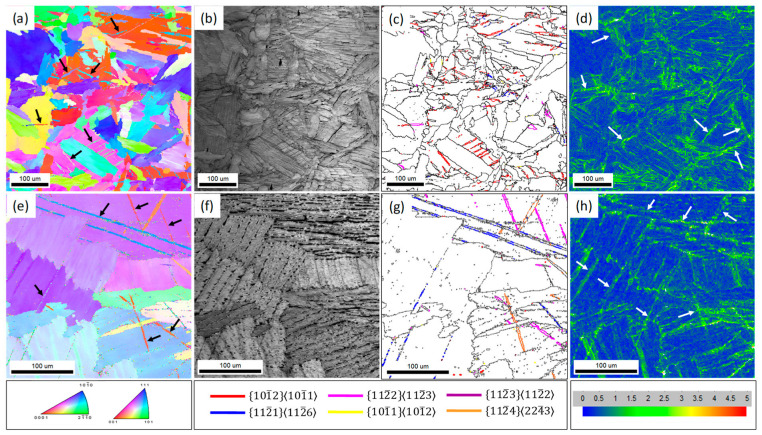
EBSD maps of MG Ti (**a**–**d**) and CG Ti (**e**–**h**) sample, about 2 mm away from the impact fracture surface. (**a**,**e**) are the inverse pole figure (IPF), (**b**,**f**) are image quality (IQ), (**c**,**g**) are the grain boundary (GB) map, and the six twinning systems are outlined in different colors, (**d**,**h**) are the local average misorientation (LAM) map.

The EBSD images of MG Ti and CG Ti after impact deformation are displayed in Figure 7. In both MG Ti and CG Ti sample, the DTs are frequently observed, as indicated by the arrows in Figure 7a,e, which is consistence with the observation of the optical microcopy. The retained β phase is too fine at a scale to be identified in the inverse pole figures (IPF), but it can be identified in the image quality (IQ), as shown in Figure 7b,f. In order to identify the DT systems, the different DTs boundaries are outlined in different colors, as shown in Figure 7c,g. For MG Ti sample, three different deformation twins, including $\left\{10\bar{1}2\right\}$, $\left\{11\bar{2}1\right\}$, and $\left\{11\bar{2}2\right\}$, are observed, and $\left\{10\bar{1}2\right\}$ is the dominated twin system, which is consistent with the TEM observation (Figure 6). For CG Ti sample, $\left\{11\bar{2}1\right\}$, $\left\{11\bar{2}2\right\},$ and $\left\{11\bar{2}4\right\}$ twin systems are observed, and $\left\{11\bar{2}1\right\}$ is the dominated twin system. Surprisingly, $\left\{10\bar{1}2\right\}$ is barely observed in the CG Ti sample even though it is considered the easiest activated twin system in hcp Ti [31,32]. It is not clear why $\left\{11\bar{2}1\right\}$ twins were frequently activated instead of $\left\{10\bar{1}2\right\}$ twins in CG Ti during impact deformation. In Figure 7d,h, the corresponding local average misorientation (LAM) maps demonstrate, for the MG Ti sample, the strain concentration mainly distributed at the grain boundaries, as indicated by the arrows in the Figure 7d. However, the strain concentration of CG Ti sample mainly distributed at the twin boundaries and α/β interface, as indicated by the arrows in Figure 7h.

## 4. Discussion

### 4.1. Deformation Mechanisms Dependent on Grain Size Distribution and Deformation Rate

**Figure 8:**
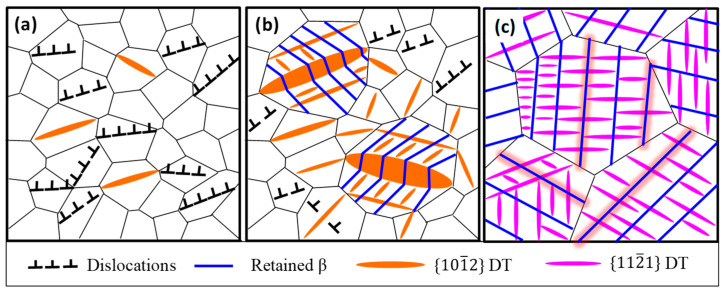
Schematic diagram of deformation modes of the different grain size characteristic TA3 alloy during impact deformation: (**a**) FG Ti, (**b**) MG Ti, and (**c**) CG Ti sample.

Based on the above microstructural observations, the deformation mechanism of different samples during impact deformation are sketched in Figure 8. It demonstrates that the deformation mechanisms are sensitive to the grain size distribution. During impact deformation, the primary deformation mode of FG Ti is dislocation slip, whereas the primary deformation modes of MG Ti are dislocation slips in fine grain region and DTs in coarse grain region. For CG Ti, DTs become the primary deformation mode (Figure 6).

As the interface between the α matrix and the retained β is not atomically flat, and comprised of a series of facets, DTs would preferentially nucleated at the α/β interface [33]. Thus, the high density of α/β interface in CG Ti provides sufficient DTs nucleation sites, resulting in the density of activated DTs in CG Ti is obviously larger than that in MG Ti and FG Ti, as displayed in Figure 5e,f. Furthermore, after impact deformation, the primary DTs are $\left\{10\bar{1}2\right\}$ for MG Ti, but they are $\left\{11\bar{2}1\right\}$ for CG Ti, as demonstrated in Figure 7. Meanwhile, the morphology of $\left\{10\bar{1}2\right\}$ twins in MG Ti is obviously wider than $\left\{11\bar{2}1\right\}$ twins in CG Ti, as shown in Figure 5. The reason why the width of $\left\{10\bar{1}2\right\}$ twins in MG Ti is larger than that of $\left\{11\bar{2}1\right\}$ twins in CG Ti is likely to be due to the different energy between nucleating a new twin in comparison with thickening its width. It is proposed that, for $\left\{10\bar{1}2\right\}$ twins in titanium, there is a lower thickening energy barrier in comparison to the energy barrier for nucleating new twins [34].

### 4.2. The Reason Why Does FG Ti Sample Exhibit the Lowest Rp_0.2_ and Highest Elongation at Break during Tensile Deformation?

According to the Hall–Petch relation, the FG Ti sample is expected to obtain the highest Rp_0.2_ and R_m_ among the FG Ti, MG Ti, and CG Ti sample. In fact, FG Ti sample exhibited the lowest Rp_0.2_. This abnormal phenomenon can be explained by the strengthening of the retained β. Due to the strong barriers of retained β to dislocation movement, the CG Ti exhibits an improved Rp_0.2_ and R_m_ even though its coarse grain would degrade its strength. This suggests that the strengthening effect of retained β is larger than the softening effect of coarse grain. For the MG Ti sample, it exhibits the highest Rp_0.2_ and R_m_ among the FG Ti, MG Ti, and CG Ti sample, which can be contributed to the coupling effect of refinement strengthening in fine grain region and retained β phase strengthening in coarse grain region. For the same reason, the maximum impact load (F_m_) of the three samples displays a similar trend as the Rp_0.2_ and R_m_. The retained β phase in MG Ti and CG Ti samples are effective obstacles to dislocation motion resulting in a higher Rp_0.2_, but they simultaneously trend to become cracking sites due to the stress concentration generated by the pileup of dislocations at α/β interface leading to a decreased A [35].

### 4.3. The Reason Why Does MG Ti Sample Exhibits the Highest E_t_ during Impact Deformation?

**Figure 9:**
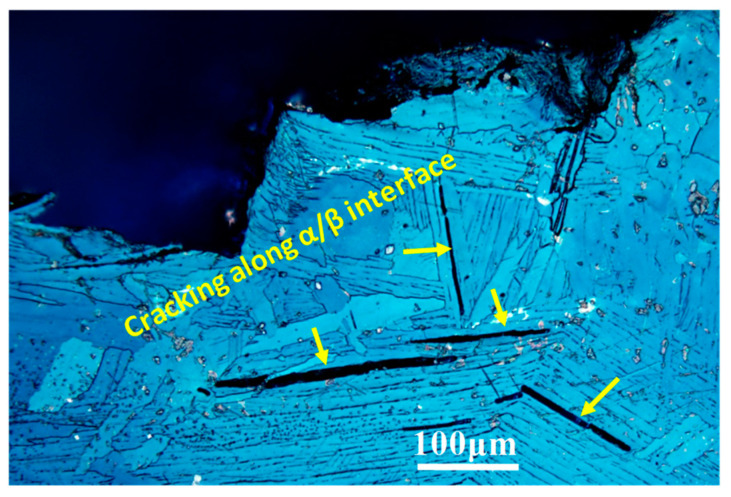
The cracks propagate along the α/β interface in CG Ti sample during impact deformation.

Normally, the impact absorbed energy is related to the tensile strength and ductility, which is proportional to the area surrounded by the tensile stress-strain curve. At present investigation, the MG Ti exhibited the largest impact absorbed energy although its area surrounded by the tensile stress-strain curve is not largest among the three samples (Figure 4). This phenomenon can be explained by the twin inducing plasticity (TWIP) effect during impact deformation. The difference of impact absorbed energy between MG Ti and CG Ti is mainly attributed to the difference of activated DTs type. Zheng et al. investigated the interaction of $\left\{10\bar{1}2\right\}$ and $\left\{11\bar{2}1\right\}$ twins with β phase, respectively. They found the β phase can occur kinking when $\left\{10\bar{1}2\right\}$ twins pass through it [36]. However, the β phase does not show plastic deformation when $\left\{11\bar{2}1\right\}$ twins pass through it [37]. In the latter case, the α/β interface will act as the strong barriers to deformation twin growth resulting in the preferential nucleation of cracks along the interface, and the corresponding crack propagation path was shown in Figure 9. Thus, compared to MG Ti sample, cracking along the α/β interface in CG Ti sample leads to decreasing of impact absorbed energy. In addition, because the width of the activated $\left\{10\bar{1}2\right\}$ twins in MG Ti sample is obviously larger than that $\left\{11\bar{2}1\right\}$ twins in CG Ti sample, the dislocation slips were easy to activate during further straining. This leads to the strain compatibility near the $\left\{10\bar{1}2\right\}$ twin boundaries is relatively better than that near the $\left\{11\bar{2}1\right\}$ twin boundaries [38], and thereby resulting in higher impact absorbed energy.

## 5. Conclusions

(1) TA3 alloy with three different grain size characteristics were produced by combing hot-rolling and annealing treatment, i.e., fine grain size (FG Ti), coarse grain size (CG Ti), and a mixture of coarse and fine grains size (MG Ti). Furthermore, a few retained β phase distributed in CG Ti and the coarse grain region of MG Ti sample.
(2) Among FG Ti, CG Ti, and MG Ti samples, MG Ti exhibits the highest tensile yield strength (Rp0.2) and impact absorbed energy, together with an intermediate tensile elongation to failure (A).
(3) During impact deformation, the primary deformation mode of FG Ti is dislocation slip, whereas the primary deformation modes of MG Ti are dislocation slips in fine grain region and $\left\{10\bar{1}2\right\}$ DTs in coarse grain region. For CG Ti, $\left\{11\bar{2}1\right\}$ DTs become the primary deformation mode.
(4) The superior combination of tensile strength and impact absorbed energy in MG Ti sample can be ascribed to the couple effect of retained β strengthening and twin inducing plasticity.

## Figures and Tables

**Table 1:** The average yield strength (Rp_0.2_) and elongation to failure (A) of the FG Ti, MG Ti and CG Ti samples.

	FG Ti	MG Ti	CG Ti
Yield strength (Rp_0.2_)/MPa	479.82	541.28	525.92
Elongation to failure (A)/%	30.44	23.93	17.23

## Data Availability

Not applicable.
